# The ups and downs of biological timers

**DOI:** 10.1186/1742-4682-2-22

**Published:** 2005-06-20

**Authors:** Noa Rappaport, Shay Winter, Naama Barkai

**Affiliations:** 1 Departments of Molecular Genetics and Physics of Complex systems, Weizmann Institute of Science, Rehovot, Israel

## Abstract

**Background:**

The need to execute a sequence of events in an orderly and timely manner is central to many biological processes, including cell cycle progression and cell differentiation. For self-perpetuating systems, such as the cell cycle oscillator, delay times between events are defined by the network of interacting proteins that propagates the system. However, protein levels inside cells are subject to genetic and environmental fluctuations, raising the question of how reliable timing is maintained.

**Results:**

We compared the robustness of different mechanisms for encoding delay times to fluctuations in protein expression levels. Gradual accumulation and gradual decay of a regulatory protein have an equivalent capacity for defining delay times. Yet, we find that the former is highly sensitive to fluctuations in gene dosage, while the latter can buffer such perturbations. In particular, a positive feedback where the degrading protein auto-enhances its own degradation may render delay times practically insensitive to gene dosage.

**Conclusion:**

While our understanding of biological timing mechanisms is still rudimentary, it is clear that there is an ample use of degradation as well as self-enhanced degradation in processes such as cell cycle and circadian clocks. We propose that degradation processes, and specifically self-enhanced degradation, will be preferred in processes where maintaining the robustness of timing is important.

## Background

Protein levels within cells are subject to genetic and environmental variations, but mechanisms have evolved that buffer cellular processes against those fluctuations [1]. Quantitative analysis has indicated that the need to ensure robustness can largely restrict the design of the underlying network [2-4]. Maintaining a reliable sequence of events appears straightforward in cases where the completion of one event directly triggers the next [5]. Often, however, temporal cascades are propagated by a self-sustained biochemical network, which functions even in the absence of feedback signals [6]. For example, the cell cycle is governed by an autonomous oscillator, although this oscillator is executively sensitive to checkpoint signals that may halt its progression [7]. Similarly, while the circadian timing is synchronized by light or temperature, it oscillates as well under constant conditions [8]. The prevalence of self-sustained networks that coordinate temporal cascades suggests that at least in certain cases not only is the temporal order important, but also the relative timing of events needs to be maintained. However, whether mechanisms that code for delay times can also buffer those times against fluctuations has not yet been examined.

**Figure 1:**
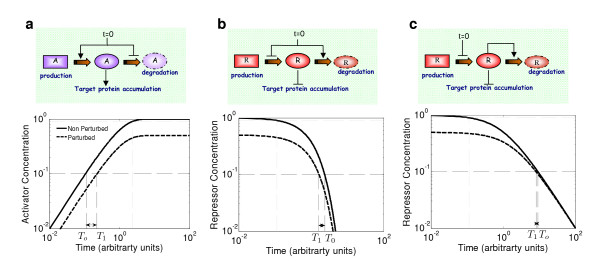
**Strategies for coding delay times**. A schematic description of each strategy is shown on the top panel, while the respective protein dynamics is shown at the bottom panel. The solid black line corresponds to some reference system, while the dashed black line corresponds to a system in which production rates were reduced two-fold. The time to reach the threshold (taken here as 10% of P_max_) is also shown. It can be seen that the delay time sensitivity is largest for accumulation and smallest for non-linear decay. Moreover, the location of the threshold is limited; threshold of 90% (light grey) will never be crossed by a perturbed system with *η *of less than 0.9. **a, **Accumulation strategy. In this case gene production is initiated at t = 0. Once a certain threshold is reached, downstream genes would be affected. **b, **Degradation strategy. In this case, protein production is stopped at t = 0. Once protein levels have decayed below a certain threshold, target genes would be affected. **c**, Same as **b, **except that degradation is non-linear with n = 2.

Strategies for coding delay times can be classified into two main categories, which are based on the accumulation or on the decay of some regulatory protein, or of its active form (Fig. 1A–B). A typical cascade employs both strategies, but it is not clear which is rate limiting for defining the timing. For example, in the budding yeast cell cycle, Cln3 accumulation is followed by Sic1 degradation, with both processes required for the G1/S transition and the initiation of START [9-12].

In this work, we compare the two strategies for coding delay times with respect to their capacity to buffer fluctuations in gene dosage.

## Results and discussion

Consider a protein *P *that is present at a low level *P*_low. _Accumulation of *P *can be initiated either by enhancing its production or by inhibiting its degradation. In either case, *P *will start accumulating toward a new steady state *P*_max_. Subsequent events will follow once P has passed some threshold *P*_*T*_, defined for example by its affinity to target genes promoters if P is a transcription activator. The corresponding delay time, T_0_, is defined by the time it takes to accumulate this threshold level *P*_*T *_of proteins. Alternatively, delay time can be encoded by an analogous system, where P decays from P_max _toward P_low_, activating subsequent events once its levels are reduced below the threshold P_*T*_.

### Comparing the robustness of time coding strategies

The above two strategies appear to be equivalent for defining delay times. For example, in the absence of feedback, where each protein is degraded independently, both accumulation and degradation follow analogous exponential profiles (Table 1). To keep the equivalence of the two profiles, we assume, for example, that the two situations are characterized by the same degradation rate *α*. Thus, the only difference between the accumulation and decay situations resides in the production rate, which is either enhanced or repressed at the onset of the respective dynamics^1^. Evidently, accumulation times are precisely the same as degradation times (Table 1); for example, the time to accumulate or degrade 90% of P_max _is given by *α*^-1^log (10). For simplicity we also assume that P_low _= 0, although our results do not depend on this assumption as long as P _low _is much lower than the threshold level P_T _(Additional file 1).

**Table 1:** Comparison of models

	Linear Model^a^		Non Linear Model^a,b^
	
	*Accumulation*	*Decay*	*Non Linear Decay*^d^
Model			*n *= 2, 3, 4...
Solution	*P *= *P*_max _(1 - *e*^*-αt*^)	*P *= *P*_max _*e*^*-αt*^	
T_0 _ Unperturbed delay time			
T_1_ Perturbed^c ^delay time	> *η*^-1^*T*_0 _ (*η *< 1) <*η*^-1^*T*_0 _ (*η *> 1)		
Delay time sensitivity	≥ |*η*^-1 ^- 1|		

^a  ^*P*_max _

^b  ^T_0_, T_1 _and *δt *are presented for n = 2

^c  ^Peturbation: *v*_0 _→ *ηv*_0_

We examined the sensitivity of the delay times to fluctuations in the production rate of P. Since production rate correlates with gene dosage, it is likely to be mostly sensitive to gene-specific perturbations. Perturbation was implemented by changing the production rate of P, *v*_0_, by some factor *η*. Consequently, the delay time T_0_, coded by the time to accumulate or degrade the protein level from its initial value to the threshold level *P*_*T *,_ is changed. We denote this perturbed time by T_1_. The sensitivity of the delay time to this change in production rate was defined by the relative change in the delay time:

            

### Delay times encoded by decay display a significantly lower sensitivity

**Figure 2:**
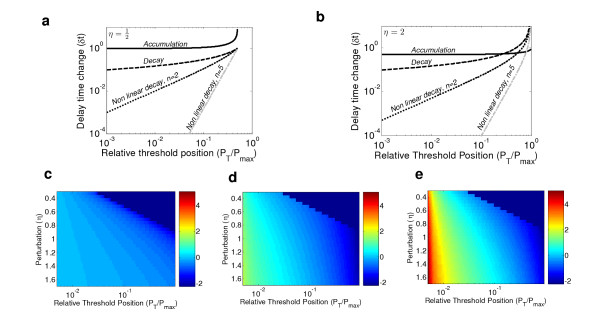
**Delay-time sensitivities for different *η *and different threshold positions**. **a**, *η *= 1/2. It can be seen that for all threshold positions the sensitivity of the delay time is smallest for non-linear decay and largest for accumulation. **b**, *η *= 2. Also here the sensitivity of the delay time is smallest for non-linear decay and largest for accumulation for most threshold positions. Note that the situation is reversed for high threshold levels corresponding to high sensitivity in all cases (fig. 1). **c-e**, Delay time sensitivity as a function of *η *and P_T _for the cases of accumulation (c), decay (d) and non-linear decay (e). The logarithm of the delay time sensitivity is shown: log (|*η *- 1|/|*δt*|) for decay and log (|*η*^-1 ^- 1|/|*δt*|) for accumulation. *δ*t was normalized by *η*-1 for decay and by *η*^-1^-1 for accumulation, which correspond to *δ*t in the non-buffered case in which T_1 _= *η*T_0 _for decay and T_1 _= *η*^-1^T_0 _for accumulation. Thus, blue represents a non-buffered system. Red represents a buffered system.

Despite their apparent equivalence, we found that the accumulation and decay strategies differ greatly in their capacities to buffer fluctuations in production rate. In fact, for most cases, delay times encoded by decay display a significantly lower sensitivity (Fig. 2 and Table 1). For example, while a two-fold reduction in production rate (*η *= 1/2) increases delay times by at least 100% in the case of accumulation, it will cause only a 15% (if P_T _= 0.01 P_max_) or 30% (if P_T _= 0.1 P_max_) decrease in the case of degradation.

The reason underlying this differential behaviour may not be immediately apparent. Within both strategies, changing protein production rate impacts the dynamics in two principal ways. First, it alters the initial rate (*v*_0_) by which a protein accumulates or degrades. Second, it modifies the maximal level *P*_max _(Fig. 1A–B). The key difference between the two systems resides in the initial conditions: in the case of accumulation, the initial condition, and thus the amount of protein that needs to accumulate in order to reach the threshold, remains fixed. Consequently, increased velocity necessarily shortens the time to reach the threshold. In contrast, in the case of degradation, the initial condition, and thus the distance to the threshold, is modified as well. Indeed, this change in initial condition partially balances the change in velocity.

Moreover, this combination of effects leads to a completely different behaviour of the delay time sensitivity, *δ*t. Whereas in the case of accumulation, perturbation in production rate can be mathematically approximated by rescaling time by a constant factor, in the case of degradation such a perturbation is captured by introducing a constant shift in time (see expressions for T_1 _in Table 1 and Additional file: 1 2.1.2). This difference is due to the fact that production rate enters the equation explicitly in the case of accumulation, but only implicitly, through the initial conditions, in the case of decay. Importantly, this distinct behaviour is not restricted to the linear model, but is in fact applicable also to a general model that includes arbitrary feedback interactions (Additional file: 1 2.2). Consequently, within the accumulation strategy, the dependence of delay times on perturbation will be at best linear with perturbation size, irrespective of possible feedbacks.

Thus, despite the apparent equivalence of the accumulation and degradation strategies, they differ greatly in their capacity to buffer delay times against perturbations in gene dosage. Still, even in the case of degradation, buffering capacity in the absence of feedbacks is limited by realistic dynamical range of the degrading protein. For example, in order to achieve <8% sensitivity to a two-fold change, protein levels have to degrade over four orders of magnitude. This need to increase the dynamical range in order to improve robustness reflects the fact that delay time sensitivity depends on the degradation rate at early times: the faster this initial decay, the greater the robustness (Additional file: 1 3.2.1). However, in the absence of feedback, the system is characterized by a uniform decay rate, so that increasing this initial degradation implies an overall faster decay of P during the given delay time.

### Non-linear degradation enhances robustness

One possible way to overcome this interplay between robustness and dynamical range is to introduce a feedback that enhances degradation specifically at early times, while maintaining moderate decay rate during the rest of the time. This will be the case, for example, if the degrading protein functions to enhance its own degradation, either directly or by changing the activity of a third protein. Indeed, a similar feedback mechanism was recently shown to enhance the spatial robustness of morphogen gradients [13].

To rigorously examine the possible impact of an auto-induced degradation on buffering capacity, we extended the linear model to include non-linear degradation (Table 1). In contrast to the exponential dynamics found in the linear system, here the system decays as a power-law in time. Examining the delay time sensitivity, we observed a significantly improved robustness (Fig. 2). For example, for moderate non-linearity, with *n *= 2, two-fold reduction in production rate will decrease the delay time by merely 1%, compared to 15% in the absence of feedback (for P_T _= 0.01 P_max,_) Moreover, increasing this coefficient of non-linearity further enhances the robustness (Fig. 2).

The robustness of timing requires fast initial degradation coupled with slower degradation afterwards. In particular, degradation needs to be rapid when protein concentration is above P_max _* *η *_min_. Nonlinear degradation enables, in principle, such flexible degradation rates. However, we note that there is an upper limit to degradation rate (see next section).

Auto-regulated degradation is implied in various stages of the cell cycle, such as the transition from S phase to mitosis and the exit from mitosis [14,15]. The degradation of the budding yeast Cdc20 exemplifies this. It begins degrading in late M phase, just before exit from mitosis, and continues throughout G1 phase [16]. This degradation is self-enhanced as Cdc20 itself is an activator of APC-dependent proteolysis through the subunits Cdc23 and Cdc27 [17]. However, degradation in those stages is commonly assumed to occupy only a small portion of the transition time, with most of the delay defined by protein accumulation. It may be that autonomous timing of those stages is less crucial, since the transitions are completely dependent on checkpoint mechanisms that survey the successful completion of the critical events occurring during those cell-cycle stages. Alternatively, protein decay may actually occupy a longer portion of the transition time, or other feedback mechanisms exist but have not yet been identified.

## *Cell degradation machinery sets a lower limit on time variability*

For robust measurement of time, the time for degradation of protein concentrations above P_max _* η _min _(denoted by δ, different from the sensitivity δt) needs to be short. However, this degradation time is bounded from below by the maximum degradation rate of the cell machinery (in this section we assume that the protein is being degraded rather than being modified):

            

*Where δ_guaranteed _is worst case δ. η_max , _η_min _are worst cases for η and deg_max _is maximum degradation rate [molecules/s]*.

*An order of magnitude estimate of this limit is given, based on a work by Shibatani and Ward *[18]*, which assayed for 20S rat proteasome activity. The 20S proteasome complex is found in all eukaryotic cells and constitutes 0.5–1% of the soluble protein in the cell.*

Shibatani and Ward have measured degradation rates in vitro, activating the proteasome with sodium dodecyl sulfate (SDS). The maximum degradation rate measured was 20 nmol/h for 0.07 nmol proteasome. I.e., each proteasome complex degraded roughly 300 molecules per hour. The proteasome composes 0.5–1% of the soluble proteins in the cell (by mass). It is a very heavy complex, about 700 kDa, 14-fold greater than average protein mass, which is roughly 50 kDa. Hence, there are about 1400–2800 proteins per each proteasome complex in the cell. Estimating protein number in the cell as 10^6^-10^7 ^(molecules) gives ~ 10^3 ^proteasome units. Each unit is capable of degrading 300 molecules in 1 hour, giving deg_max _~ 100 [proteins/s].

Assuming P_max _~ 10^3 ^molecules, and fluctuations of the same order (e.g., η = 2), δ_guaranteed _= 10 seconds. Processes sufficiently longer than δ_guaranteed _can be measured accurately: even relaxing some of the assumptions (degradation dedicated to single protein, larger P_max_etc.), will enable timing of many processes with good accuracy. For example, yeast cell cycle is about 120 minutes, circadian clock is 24 hours – 10^3^-10^4 ^fold longer than δ_guaranteed._

### Perturbation to production vs. degradation rates

Our discussion focused on the robustness to fluctuations in production rate (*v*_o_) while assuming degradation rate (*α*) to be relatively stable. Since the degradation machinery plays a crucial role in numerous cellular processes, it is reasonable to assume that its abundance is under a tight regulation, which also limits the noise in degradation rates of individual proteins. Moreover, gene dosage perturbations to production rate are of large magnitude compared to other sources of noise. We expect, as consequence, that mechanisms for buffering against production rate perturbations will be abundant.

Different buffering mechanisms will need to be utilized in the alternative situations where fluctuations in degradation rate dominate. One possible scheme that could reduce the effect of fluctuations in degradation relies on *in-cis *degradation, where each molecule promotes its own degradation. Such a mechanism was recently reported in the context of cell-cycle timing, where S-phase can only start after UbcH10 undergoes *in-cis *degradation [19]. Alternatively, delay time could be coded by the linear phase of accumulation, before degradation comes into effect. In this case, the delay time is given by T_o _= P_T_/*v*_*o *_and does not depend on the degradation rate.

More generally, one may envision other noise characteristics, each dictating its own limitations; for example both production rate and degradation rate might be perturbed together (e.g. temperature effect). The threshold P_T _might be perturbed together with production rate (Additional file: 1 7) or any other perturbation characteristics. Different buffering mechanisms may need to be tuned for these different perturbations types, which could be analyzed using the framework presented in this paper.

## Conclusions

Ensuring the robustness of timing may be of particular importance in order to support crosstalk amongst several processes that are executed in parallel. In such cases, not only the successful completion of events, but also maintaining the coordination, is important. This need may be of particular relevance during development of multicellular organisms, where multiple differentiation processes often proceed in parallel. Our identification of mechanisms that are able to maintain such robustness of timing may provide a new framework for examining the robustness of the long-range cascades that underlie those processes.

Our discussion focused on timing mechanisms that rely on the accumulation or degradation of a single protein component. While such mechanisms can serve as independent timers, more often they present an elementary unit in a more complex cascade. For example, models of cell cycle regulation propose that delay times are generated through the activation of some intermediate components, leading to a delayed negative feedback [20,21]. Further work is required to define how the properties of the full cascade are determined from the properties of its elementary units, and what additional constraints are required for proper coupling of different elementary units.

## Methods

Figures were generated using Matlab simulations.

## Competing interests

The author(s) declare that they have no competing interests.

## Authors' contributions

NR performed the analysis and drafted the manuscript. SW performed the analysis and drafted the manuscript. NB conceived of the study, participated in its design and drafted the manuscript. All authors read and approved the final manuscript.

## Note

^1 ^The results are unchanged if we keep production rate fixed and vary the degradation rate. This will become clear later (see expressions for delay time sensitivity in table 1).

## Supplementary Material

Additional File 1 Supplementary Information Click here for file
