# Supplementary material for: The ups and downs of biological timers
Source: Theor Biol Med Model. 2005 Jun 20;2:22. doi: 10.1186/1742-4682-2-22 (PMC1208956; doi:10.1186/1742-4682-2-22)
Supplement: Additional File 1 — Supplementary Information [file 1742-4682-2-22-S1.pdf]

The Ups and Downs of Biological Timers

Supplementary Information

Noa Rappaport, Shay Winter, Naama Barkai*

Departments of Molecular Genetics and Physics of Complex Systems
Weizmann Institute of Science
Rehovot, Israel

March 31, 2005

* Corresponding author: E-mail: naama.barkai@weizmann.ac.il , *Telephone: +972-8-9344429* ,

Fax: +972-8-9344108

Contents

1. Delay time coding strategies 3
2. Accumulation 3
2.1. Linear case 3
2.1.1. Approximation 4
2.1.2. Perturbation effect 4
2.2. General case 5
2.2.1. Perturbation effect 5
3. Decay 6
3.1. Linear case 6
3.1.1. Perturbation Effect 7
3.2. General Case 7
3.2.1. Perturbation effect 8
4. Non–linear degradation 9
4.1. Perturbation effect 10
5. Generalization for the case of $P_{initial}$ and P_{final} different than zero 10
5.1. Accumulation 10
5.1.1. $\eta<1$ 11
5.1.2. $\eta>1$ 12
5.2. Decay 12
5.2.1. Both assumptions are valid 13
5.2.2. The assumption $P_i \gg P_f$ is not valid 13
5.2.3. The assumption $P_i \gg P_f$ is valid, but the assumption $P_T \gg P_f$ is not valid 13
6. Differences between modifications and expression / degradation 14
7. Simultaneous perturbation to production rate and threshold level 15

1. Delay time coding strategies

Delay time coding can typically be obtained by:

1. **Accumulation of a protein** by de novo transcription, inhibition of degradation, or analogously by a modification of a non-active form or inhibition of modification of the active form.
2. **Gradual decay of a protein** either by halting production, increasing degradation or analogously increasing modification of the active form or decreasing modification of the non-active form.

In both cases the protein will serve to induce\repress transcription\modification of a downstream-regulated protein, once crossing activation threshold.
Here we provide the complete solution for these two cases and analyze their sensitivity and robustness to fluctuation in the protein production rate.

2. Accumulation

2.1. Linear case

Let's suppose we have a protein P and assume, without loss of generality, it is an activator. In order to induce transcription of a downstream protein, P must accumulate above a level P_T. In the absence of feedbacks, the dynamics would be described by the following equation:

$$\frac{dP}{dt} = v_0 - \alpha P \tag{1}$$

This equation simply assumes the rate of change of the protein level is affected by its production rate v_0 on the one hand, and the degradation rate α on the other hand, assuming each protein molecule decays independently. This equation can be solved analytically to yield:

$$P = P_{\max} + (P_{low} - P_{\max})e^{-\alpha t} \tag{2}$$

With:

$$P_{\max} = \frac{v_0}{\alpha} \tag{3}$$

This is an exponential profile, in which α sets the time-scale of accumulation. Therefore, the time T_0 it takes P to get to a threshold level P_T starting from initial concentration P_{low} is:

$$T_0 = \frac{1}{\alpha}\ln\left(\frac{P_{low} - P_{\max}}{P_T - P_{\max}}\right) \tag{4}$$

For simplicity, we will assume the initial P is zero (see section 5 for generalization to $P_{low} \neq 0$). Therefore, we get:

$$T_0 = \frac{1}{\alpha}\ln\left(\frac{P_{\max}}{P_{\max} - P_T}\right) \tag{5}$$

2.1.1. Approximation

Under the linear order approximation:

$$\alpha t << 1 \tag{6}$$

We can estimate:

$$e^{-\alpha t} = 1 - \alpha t \tag{7}$$

Thus we get:

$$P \approx P_{\max}\alpha t \tag{8}$$

And:

$$T_0 = \frac{1}{\alpha}\frac{P_T}{P_{\max}} \tag{9}$$

2.1.2. Perturbation effect

Gene dosage variations as well as perturbations of the protein production rate, will have an effect through v_0:

$$v_0 \rightarrow \eta v_0 \tag{10}$$

This perturbation will affect the protein's initial rate of accumulation and will also linearly affect the steady state level of the protein, P_{max}.
Note that in order to ensure the existence of a solution ($P = P_T$) also for the perturbed system, we need to demand:

$$P_T < \eta P_{\max} \tag{11}$$

For example, in Figure 1 it can be seen in light grey that a threshold position of $P_T = 0.9$ will not be reached if η= ½, since the new steady state level will be lower than the threshold.
We are interested in the relative change of the delay time in the perturbed system.
Let us define T_1 as the time it takes P to reach the same threshold level P_T in the perturbed system. Thus:

$$T_1 = \frac{1}{\alpha}\ln\left(\frac{1}{1-\frac{P_T}{\eta P_{\max}}}\right) \tag{12}$$

And under the linear approximation (Eq. (9)):

$$T_1 = \frac{1}{\alpha}\frac{P_T}{\eta P_{\max}} \tag{13}$$

Therefore, the delay time sensitivity, δt, will be:

$$\delta t_{acc} = \left|\frac{T_1 - T_0}{T_0}\right| = \frac{\ln\left(\frac{\eta - b}{\eta(1-b)}\right)}{\ln(1-b)} \tag{14}$$

While:

$$b = \frac{P_T}{P_{\max}} \tag{15}$$

For the linear approximation, we get the following expression:

$$\delta t_{acc} = \left|\eta^{-1} - 1\right| \tag{16}$$

2.2. General case

In this section we will show that at best, delay time scales linearly with the perturbation factor, and generally it is even more sensitive.
Let's suppose we have a protein P. Its dynamics can be described by the following equation:

$$\frac{dP}{dt} = v_0(P) - F(P) \tag{17}$$

For the equation to describe accumulation, the following demands have to be fulfilled:

$$\begin{aligned} &v_0(P) - F(P) > 0 \\ &F(P) > 0 \end{aligned} \tag{18}$$

Note that the second condition in Eq. (18) does not limit generality of model, if production rate is bounded.

2.2.1. Perturbation effect

Perturbation of the gene production rate (such as gene dosage effect) can be modeled by a perturbation of v_0 to yield:

$$\frac{d\tilde{P}}{dt} = \eta v_0(\tilde{P}) - F(\tilde{P}) \tag{19}$$

Similarly, there is a demand on the perturbation level to be large enough to allow accumulation of the protein:

$$\eta v_0(P) - F(P) > 0 \tag{20}$$

Thus, after a simple two-sided integration we get:

$$T_0 = \int_0^{P_T} \frac{dP}{v_0(P) - F(P)} \tag{21}$$

$$T_1 = \int_0^{P_T} \frac{dP}{\eta v_0(P) - F(P)} \tag{22}$$

And therefore the normalized delay change will be:

$$\delta t_{acc} = \left| \frac{T_1 - T_0}{T_0} \right| = \left| (\eta^{-1} - 1) \frac{\int_0^{P_T} \frac{dP}{\left(1 - \frac{F(P)}{v_0(P)}\right)\left(1 - \frac{F(P)}{\eta v_0(P)}\right)}}{\int_0^{P_T} \frac{dP}{\left(1 - \frac{F(P)}{v_0(P)}\right)}} \right| \tag{23}$$

Both integrals sum over same section [0, P_T], but since $\left| 1 - \frac{F(P)}{\eta v_0} \right| < 1$ (see Eq.(18) and Eq. (20)), the nominator integrand is bigger than that in the denominator. Therefore, the ratio between the two integrals is greater than 1. Thus, we get:

$$\delta t_{acc} \geq \left| \eta^{-1} - 1 \right| \tag{24}$$

This proves that in the general accumulation case, the delay time sensitivity δt_{acc} is always worse than a linear dependence on the perturbation size. Moreover, the smallest error in case of accumulation is received in the linear order of a simple linear case (when the system is far from saturation).

3. Decay

3.1. Linear case

In the case of a decaying protein, at t=0 the production of the protein is effectively stopped, and therefore the protein level decays to a new steady state level, which is

close to zero. Without loss of generality, we'll assume the protein is a repressor which stops repression of a downstream gene once it degrades bellow the repression threshold. We can estimate the equation by:

$$\frac{dP}{dt} = -\alpha P \tag{25}$$

$$P_{(t=0)} = P_{\max} = \frac{v_0}{\alpha}$$

We assume the protein starts decaying from a steady state level P_{max}, which is actually the maximum level reached by the protein following induction (see previous section). This equation can easily be solved to yield:

$$P = P_{\max} e^{-\alpha t} \tag{26}$$

Therefore, the time T_0 in which P degrade to a threshold level P_T is:

$$T_0 = \frac{1}{\alpha} \ln\left(\frac{P_{\max}}{P_T}\right) \tag{27}$$

3.1.1. Perturbation Effect

In an analogous way, perturbation will cause:

$$v_0 \rightarrow \eta v_0 \tag{28}$$

Therefore, the time it takes the perturbed system to get a threshold protein level is:

$$T_1 = \frac{1}{\alpha} \ln\left(\frac{\eta P_{\max}}{P_T}\right) \tag{29}$$

Therefore, the delay time sensitivity δt (defined in the same way as in the case of accumulation) will be:

$$\delta t_{dec} = \frac{\ln(\eta)}{\ln\left(\frac{P_{\max}}{P_T}\right)} \tag{30}$$

This dependency of the delay time sensitivity on the perturbation is much milder than the dependency in the accumulation case, as it is logarithmic rather than linear (as in the best case of induction). It is also evident that by increasing the ratio P_{max}/P_T we can decrease the error. This improvement of accuracy is primarily restricted by the maximal possible ratio, the dynamical range of the system.

3.2. General Case

For the general case of repression, in t<0 we have:

$$\frac{dP}{dt} = v_0(P) - F(P) \tag{31}$$

So that at t=0, P has already reached the steady state level P_{max} and thus:

$$F(P_{\max}) = F(P_{(t=0)}) = v_0(P) \tag{32}$$

We assume that at time t=0 production effectively ceases and we have:

$$\frac{dP}{dt} = -F(P) \tag{33}$$

3.2.1. Perturbation effect

In the general case of decay, an analogous perturbation will affect v_0, which will affect only the initial condition, but not the equation itself . Therefore in this case a perturbation can be captured by a shift in the temporal axis. Specifically, let $\tilde{P}$ be the protein level in the perturbed system and P the protein level in the non-perturbed system, then we obtain:

$$\tilde{P}(t) = P(t+\delta) \tag{34}$$

With δ defined according to:

$$\tilde{P}(0) = P(\delta) = \frac{\eta v_0}{\alpha} \tag{35}$$

For the perturbed level $\tilde{P}$, we get analogously that:

$$F(\tilde{P}_{\max}) = F(\tilde{P}_{(t=0)}) = \eta v_0(P) \tag{36}$$

And:

$$\frac{d\tilde{P}}{dt} = -F(\tilde{P}) \tag{37}$$

Under the assumption that δ is small, and that in its vicinity F(P) doesn't change fast, we can solve for δ to get:

$$P'\big|_{P=P_{\max}} = \frac{\Delta P}{\delta} = \frac{P_{\max}(\eta-1)}{\delta}$$

$$P'\big|_{P=P_{\max}} = -F(P_{\max})$$

$$\delta = \frac{P_{\max}(1-\eta)}{F(P_{\max})}$$

The quantity P'/P is commonly known as characteristic time (or length in case of spatial gradients).
In the linear case:

$$\delta = \frac{1-\eta}{\alpha} \tag{38}$$

The following conclusions can be drawn:

1. The size of the delay, and thus the robustness, is determined by the initial decay rate.
2. The total decay time depends on the average decay rate through the whole range.
3. The delay time does not depend on the threshold location.
4. For decay, a threshold that will be far enough from the initial point will be less sensitive to perturbations, while we demonstrated that this is not the case for the accumulation case.

Therefore, a possible optimization in order to get robustness will involve:

1. Fast decay near t=0, to buffer the perturbation which is done in the initial level.
2. Slower decay later, in order to spend most time at the low, unperturbed protein levels.

However, the above requirements cannot be fulfilled by an exponential profile, which has a constant decay rate. We now look into an alternative profile which allows for de-coupling of the initial and later degradation rates.

4. Non–linear degradation

A power law profile can allow for a high initial decay rate as well as a slow decay rate in later times, and therefore can de-couple the need for fast initial decay and a long total decay time, unlike an exponential decay.
Suppose we describe the non-linear degradation through the following equation of a protein which starts decaying from a level of P_{max}:

$$\frac{dP}{dt} = -\alpha P^n, \qquad n = 2,3,4... \tag{39}$$

This equation can be solved to yield:

$$P = \frac{A}{(t+\varepsilon)^m} \qquad m = \frac{1}{n-1} \qquad A = \left(\frac{m}{\alpha}\right)^{1/n-1} \tag{40}$$

The initial conditions affect the solution through ε by:

$$\varepsilon = \left(\frac{A}{P_0}\right)^{1/m} = \frac{1}{(n-1)\alpha P_0^{n-1}} \tag{41}$$

ε can be made as small as desired by controlling P_0, α or n.
For n=2 we get:

$$P = \frac{P_{\max}}{\alpha P_{\max} t + 1} \tag{42}$$

Thus, the time to get to the threshold is:

$$T_0 = \frac{P_{\max} - P_T}{\alpha P_T P_{\max}} \tag{43}$$

4.1. Perturbation effect

Following perturbation, the delay time will change to:

$$T_1 = \frac{\eta P_{\max} - P_T}{\eta \alpha P_T P_{\max}} \tag{44}$$

And the delay time sensitivity will be:

$$\delta t = \left| \frac{P_T / P_{\max}}{P_T / P_{\max} - 1} \left(\eta^{-1} - 1 \right) \right| \tag{45}$$

The delay time sensitivity decreases linearly with P_T/P_{max}, which is much faster than the logarithmic improvement in linear decay. This is manifested in figure 1C. This effect becomes stronger for larger n (figure 1C and 2A-B).

5. Generalization for the case of $P_{initial}$ and P_{final} different than zero

5.1. Accumulation

Let us define:

$$\begin{aligned} P_i &= P(t=0) \\ P_f &= P(t \to \infty) \end{aligned} \tag{46}$$

For accumulation, we will have $P_i < P_f$.

In this case we can intuitively see ,for example, that both increasing the initial rate of production by the perturbation as well as elevating the initial level of the protein contribute to shorten the delay time and thus introducing a basal level of protein will always harm the robustness.

We can show in the same rigorous way as in 2.2 that the delay time sensitivity is at best linear with the perturbation.

Analogously to Eq. (21)-(22) we get:

$$T_0 = \int_{P_i}^{P_T} \frac{dP}{v_0(P) - F(P)} \tag{47}$$

$$T_1 = \int_{\eta P_i}^{P_T} \frac{dP}{\eta v_0(P) - F(P)} \tag{48}$$

Assuming that the initial level is affected by the same perturbation factor.
Therefore, the delay time sensitivity is:

$$\delta t_{acc} = \left| \frac{\int_{\eta P_i}^{P_T} \frac{dP}{\eta v_0(P) - F(P)} - \int_{P_i}^{P_T} \frac{dP}{v_0(P) - F(P)}}{\int_{P_i}^{P_T} \frac{dP}{v_0(P) - F(P)}} \right| \tag{49}$$

5.1.1. η<1

Let's denote by φ the following expression, which is similar to the integral part of the expression we got for the delay time sensitivity in Eq. (23) :

$$\varphi = \left| \frac{\int_{P_i}^{P_T} \frac{dP}{\left(1 - \frac{F(P)}{v_0(P)}\right)\left(1 - \frac{F(P)}{\eta v_0(P)}\right)}}{\int_{P_i}^{P_T} \frac{dP}{\left(1 - \frac{F(P)}{v_0(P)}\right)}} \right| \tag{50}$$

Analogously, we conclude that φ>1.

After some calculations we get that:

$$\delta t_{acc} = \left| \left(\eta^{-1} - 1\right)\varphi + \frac{\int_{\eta Pi}^{P_i} \frac{dP}{\eta v_0(P) - F(P)}}{\int_{P_i}^{P_T} \frac{dP}{v_0(P) - F(P)}} \right| \tag{51}$$

Since the integrands are positive (Eq. (18) and Eq. (20)) , the integral borders are monotonic and $\left(\eta^{-1} - 1\right)\varphi > 0$, we get that the integral addition is positive, and thus, also in this case we prove that:

$$\delta t_{acc} \geq \left|\eta^{-1} - 1\right| \tag{52}$$

5.1.2. η>1

After some calculations we get:

$$\delta t_{acc} = \left| \left(\eta^{-1} - 1\right)\varphi - \eta^{-1} \frac{\displaystyle\int_{Pi}^{\eta P_i} \frac{dP}{1 - \frac{F(P)}{\eta v_0(P)}}}{\displaystyle\int_{P_i}^{P_T} \frac{dP}{1 - \frac{F(P)}{v_0(P)}}} \right| \quad (53)$$

Since in this case $\left(\eta^{-1} - 1\right)$ is negative, φ > 1 and the integrals ratio is positive, we get that:

$$\delta t_{acc} \geq \left|\eta^{-1} - 1\right| \quad (54)$$

5.2. Decay

For decay we will have $P_i > P_f$.

In the general case of decay, where we don't assume the protein decays to zero, the following equation applies:

$$\frac{dP}{dt} = v_0 - \alpha P \quad (55)$$

The general solution for this equation is:

$$P = P_f + (P_i - P_f)e^{-\alpha t} \quad (56)$$

The time T_0 to get to a threshold level P_T is:

$$T_0 = \frac{1}{\alpha}\ln\left(\frac{P_i - P_f}{P_T - P_f}\right) \quad (57)$$

Now, assuming that a perturbation affects not only the initial condition, but also the final steady state level by the same factor η, we get:

$$T_1 = \frac{1}{\alpha}\ln\left(\frac{\eta\left(P_i - P_f\right)}{P_T - \eta P_f}\right) \quad (58)$$

Which results in the following delay time sensitivity (with b defined as $\frac{P_T}{P_f}$,

analogously to Eq. (14)):

$$\delta t_{dec} = \frac{\ln\left(\frac{\eta - b}{\eta(1-b)}\right)}{\ln(b-1) - \ln\left(\frac{P_i}{P_f} - 1\right)} \tag{59}$$

Algebraic manipulations show that this equation can be approximated to Eq. (30), under the following assumptions:

$$P_i \gg P_f \tag{60}$$

$$P_T \gg P_f \tag{61}$$

$$P_T \gg \eta P_f \tag{62}$$

Eq. (61)-(62) actually describe the same assumption, as η is roughly at the order of magnitude of 1.
Therefore, we can divide into three cases, which will be described in the following sections. Notice that if $P_T \gg P_f$ it entails that $P_i \gg P_f$ and therefore both assumptions are valid.

5.2.1. Both assumptions are valid

In this case, the expression we got in Eq. (30) holds, and same conclusions can be drawn regarding the robustness.

5.2.2. The assumption $P_i \gg P_f$ is not valid

In this case there is no way to make a robust measurement of timing in the system since both initial and final levels are close and linearly affected by the perturbation.

5.2.3. The assumption $P_i \gg P_f$ is valid, but the assumption $P_T \gg P_f$ is not valid

In this case, equation (59) can be approximated by the following expression:

$$\delta t = \frac{\ln\left(\frac{1-b}{1-\frac{b}{\eta}}\right)}{\ln\left(\frac{P_i}{P_T - P_f}\right)} \tag{63}$$

Robust timing can be achieved in this system by tuning P_T. While the term in the nominator cannot blow as η is in the order of magnitude of 1, the term in the

denominator can be made very large (and thus decreasing δt) by tuning P_T to be very close to P_f. However, the possibility to decrease P_T is limited by the scale of noise in P_f.

6. Differences between modifications and expression / degradation

It is well known that a large portion of the cellular signaling is done by protein-protein modification. This case is different than the case of one gene which turns on the production of the following gene in the sense that in this case we have a constant total protein level, while part of it is in the activated form and the other part is in the non-activated form, but their sum is constant.

$$P \underset{v_1}{\overset{v_0}{\rightleftharpoons}} P^*$$

$$P^* + P = P^{tot}$$

We want to check whether this system is robust to fluctuation in P^{tot}. In this scheme, the two velocities, v_0 and v_1, are setting our timescale, and we assume them to be tightly controlled by the cell.

As opposed to protein expression and degradation, this situation is symmetric – while one form accumulates, the other undergoes decay. Therefore we'll divide it into different cases:

1. **Total modification** when either $v_0 = 0$ or $v_1 = 0$ (see Eq. (65)).
2. **Modification to intermediate level** when both velocities are significantly different than zero.

We can solve:

$$\frac{dP}{dt} = -v_0 P + v_1 P^* = -v_0 P + v_1 (P^{tot} - P) = v_1 P^{tot} - (v_0 + v_1) P \tag{64}$$

Note that we got the familiar linear degradation-accumulation profile. Note that decay coefficient, v_0+v_1, is assumed to be tightly controlled by the cell, while accumulation rate is linearly dependant on P^{tot}. Whether P will decay by modification into a lower level, or accumulate to a higher level, depends on initial P, denoted as P_i, and steady state level, given by:

$$\begin{aligned} P_{steady-state} &= P_f = P^{tot} \frac{v_0}{v_0 + v_1} \\ P_f^* &= P^{tot} \frac{v_1}{v_0 + v_1} \end{aligned} \tag{65}$$

For total modification, the cell can monitor the level of the decaying form, therefore achieving parallel characteristics to protein degradation to zero. In this case, all our results so far for degradation apply: robustness against perturbations can be achieved by

setting threshold near zero and even farther enhanced by using non-linear modification profile, where the decaying form enhances its own decay (which is analogous to the accumulated one represses its own accumulation).

If the final level is significantly different then zero, it will be sensitive to P^{tot} (Eq.(65)), therefore preventing us from setting threshold near it, quite similar to accumulation case (where we cannot set a threshold near P^{max} because it fluctuates). The same results of protein degradation to non-zero level apply here - if the final level (P_f) is of same order as P^{tot}, we cannot buffer against its fluctuations.

To summarize, in order to make robust time measuring system by protein modification, it needs to fully modify to one of the two forms, and measure the level of the other, decaying, form.

7. Simultaneous perturbation to production rate and threshold level

Up till now we assumed that the threshold is fixed, and only protein levels are perturbed. One may envision different case, where threshold is perturbed together with production rate – for example, due to some global cause, such as temperature change, or due to internal compensation of the system. We stress that such perturbation is unlikely, since threshold levels are generally set by other proteins and mechanisms and not by the proteins that code for the delay time itself. Hence, threshold perturbations are not expected to be correlated with perturbations of production rate of the protein that undergoes accumulation or decay. Nevertheless, we follow this line of thought in this section.

We model this simultaneous perturbation by changing $v_0 \rightarrow \eta v_0$ & $P_T \rightarrow \eta P_T$. This perturbation does not affect timing both for linear accumulation and for linear decay, since it cancels out (see equations (4) and (27)) – delay time depends on the ratio between concentrations to threshold. Nonlinear decay, however, is sensitive to this kind of perturbation, and its sensitivity grows as the nonlinearity grows. Recall expression for T_0 (eq. (43)), we'll rewrite for general nonlinearity coefficient:

$$T_0 = \alpha^{-1}\left(P_T^{-(n-1)} - P_{\max}^{-(n-1)}\right) \tag{66}$$

Applying perturbation to threshold and protein level simultaneously will rescale time by $\eta^{-(n-1)}$. Delay time sensitivity:

$$\delta t = \left|\frac{1}{\eta^{n-1}} - 1\right| \tag{67}$$

To conclude, linear accumulation and linear degradation are insensitive to this type of perturbation, while nonlinear decay is sensitive. Therefore, in case the system has internal capability to compensate for production rate change by changing threshold, both linear dynamics will be preferred, robustness speaking, over nonlinear schemes.
